# Challenges and Strategies for Enhancing Nurse Managers’ Financial Management Competencies: A Scoping Review

**DOI:** 10.1155/jonm/7525983

**Published:** 2026-05-23

**Authors:** Jiae Lee, Yoonjung Kim, Eunjin Kim

**Affiliations:** ^1^ Department of Nursing, College of Medicine, Catholic Kwandong University, Gangneung, South Korea, cku.ac.kr; ^2^ School of Nursing, Hallym University, Chuncheon, South Korea, hallym.or.kr; ^3^ College of Nursing, Ewha Womans University, Seoul, South Korea, ewha.ac.kr

**Keywords:** cost control, financial management, health care costs, health care economics and organizations, hospital administration, leadership, nurse administrators

## Abstract

**Background:**

As the financial responsibilities of nurse managers continue to grow in today’s rapidly changing healthcare landscape, there is a clear need to strengthen their financial management competencies. These competencies have historically received less emphasis in the managerial role description compared to other leadership skills.

**Aim:**

This scoping review aimed to identify factors that hinder nurse managers’ financial management and practical approaches to improve financial management for future implementation in nursing administration practice.

**Methods:**

We used Arksey and O’Malley’s five‐stage framework to structure and guide the scoping review. A systematic search strategy was implemented using the databases PubMed, CINAHL, PsycINFO, and Scopus, with the literature search completed on November 22, 2024.

**Results:**

Of the 2142 records retrieved, 14 studies were included in the review, with a total of 1518 participants across all study samples. Most of the studies (64.2%) were conducted in the USA. Thematic analysis identified three major challenges to effective financial management among nurse managers: (1) insufficient awareness, (2) limited authority, and (3) inadequate resources and systems. To overcome these challenges, three key strategic approaches to improve financial management were recognized: (1) practical experience, (2) professional credentials, and (3) targeted training and educational programs.

**Conclusion:**

The findings underscore the critical role of both institutional structures and tailored educational supports in enabling nurse managers to effectively carry out their financial duties. Specifically, strengthening competencies requires aligning managerial authority with responsibilities, improving access to systems and resources, and promoting experiential learning, credentialing, and targeted training. These insights offer practical and policy implications for strengthening competencies within nursing leadership.

**Implications for Nursing Management:**

The findings suggest that healthcare organizations should prioritize policy and structural changes to support nurse managers’ financial roles, including expanding managerial authority and strengthening support systems. Such efforts may enhance leadership capacity and improve financial performance in healthcare.

## 1. Introduction

Healthcare environments are complex and dynamic, characterized by financial instability, and faced with external pressures such as healthcare reform, regulatory requirements, and technological advancements [1, 2]. In response, hospital organizations seek strategies such as cost efficiency, resource optimization, and operational innovation [1, 2]. In today’s healthcare landscape, nurse managers are assuming a more significant leadership role beyond staffing and task management. They also act as financial managers, contributing to ensuring the financial soundness of nursing units and improving the hospital’s profitability [3]. By implementing effective inventory control and engaging in cost‐containment strategies, such as staffing optimization and waste reduction, nurse managers contribute to improved financial performance at both the unit and organizational levels [4].

Hospital financial performance is closely linked to quality of care, as demonstrated by reduced readmission and mortality rates in key patient groups [5]. Active engagement of nurse managers in budget planning, implementation, and monitoring empowers them to advocate for patient needs while aligning nursing operations with hospital financial goals [6]. Effective decision‐making is recognized as a key competency influencing the quality of nursing care, and prior evidence suggests that enhancing specific nursing competencies positively contributes to decision‐making ability [7, 8]. Given that nurse managers are responsible for various managerial decisions, including financial planning and resource allocation, financial management competency can be considered an essential component of managerial decision‐making [4]. Therefore, strengthening nurse managers’ financial management competencies is essential to support organizational objectives alongside optimal patient care [4, 9].

However, the development of nurse managers’ financial management competencies has been hindered by staff shortages, cost‐cutting measures for essential supplies, limited motivation, poor interdepartmental communication, and challenges related to budget constraints [3, 10]. Instead, it has been proposed that a way to better understand nurses’ financial behaviors is to record their activities alongside department or organizational financial indicators [3]. This suggests that nurse managers struggle to manage financial data. Yet, to date, no study has systematically mapped the full scope of challenges nurse managers face and the strategies they employ to develop financial management competencies.

This study aims to fill this gap by conducting a scoping review, which is particularly suited for mapping evidence across a broad, heterogeneous body of literature where the topic is still emerging and a systematic review would be premature [11]. This review comprehensively explores the obstacles nurse managers face and the strategies they employ to improve their financial management.

## 2. Methods

This scoping review followed the five‐stage framework established by Arksey and O’Malley [12] and is reported in accordance with the PRISMA‐ScR (Preferred Reporting Items for Systematic reviews and Meta‐Analyses extension for Scoping Reviews) guidelines [13]. The five stages were (1) formulating the research question, (2) identifying relevant studies, (3) selecting the relevant literature, (4) extracting and charting the data, and (5) collating, summarizing, and disseminating the findings.

### 2.1. Formulating the Research Question

The main research question for this study is “What are the challenges to effective financial management among nurse managers?” This question seeks to explore factors hindering nurse managers’ financial management.

The secondary research question is “What strategies are available to enhance financial management among nurse managers?” This question aims to discover practical solutions that can be implemented.

### 2.2. Identifying Relevant Studies

We used the PRISMA 2020 guidelines in the search phase of this review for a thorough and targeted literature identification process [14]. Searches were conducted in PubMed, CINAHL, PsycINFO, and Scopus using combinations of the following search terms, their synonyms, and corresponding subject headings: (“first line manager” or “head nurses” or “nurse managers” or “nurse administrators” or “charge nurses” or “supervisory nursing” or “nurse executives”), and (“budget∗” or “financial” or “payroll” or “charg∗” or “fees”). Boolean operators (AND, OR) and truncation (∗) were applied throughout, and search strategies were adapted to the syntax and subject heading conventions of each database, including MeSH terms in PubMed and Subject Headings in CINAHL. Gray literature was not searched, as this review was limited to peer‐reviewed empirical studies to ensure methodological rigor and comparability of findings. The search was completed on November 22, 2024, and the full search strategies for each database are provided in the Supporting Information.

### 2.3. Selecting Relevant Literature

We included studies that addressed financial management by nurse managers. Studies were excluded if (a) they did not target nurse managers related to financial management, (b) we were unable to determine the financial management results for the managers only, (c) they were review papers, case reports, editorials, letters to the editor, dissertations, book chapters, newspaper articles, or abstract‐only papers, or (d) we could not obtain full text.

**FIGURE 1:**
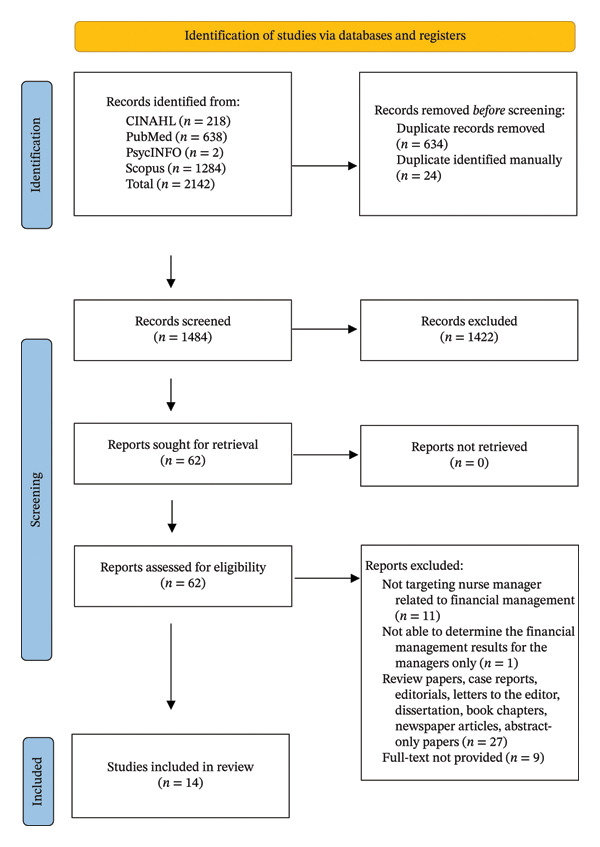
Study selection process: PRISMA 2020 flow diagram.

All three authors contributed to the screening and review process. Two authors (JL and EK) independently conducted the title and abstract screening as well as the full‐text review using Covidence software, while the third author (YK) was available to resolve any discrepancies. Inter‐rater agreement was not formally calculated; however, any discrepancies identified during the screening process were resolved through discussion and consensus among the authors. The initial literature search yielded 2142 articles. After removing duplicates and screening titles and abstracts, 62 articles were selected for the full‐text review. Ultimately, 14 studies met the inclusion criteria and were incorporated into the final scoping review (Figure 1).

### 2.4. Extracting and Charting the Data

We conducted a detailed review of the included articles and recorded relevant information using a data‐charting form developed by the research team based on the review objectives. The chart captured details such as authors, publication year, country, study design, participants, and main relevant findings (Table 1). In this review, the term “nurse manager” is used broadly to encompass the various managerial roles reported in the original studies, which included nurse unit managers (clinical nurse managers), nurse executives, directors of nursing (DONs), and other supervisory positions.

**TABLE 1:** Summary of studies included in the scoping review.

No.	Author (Year)	Country	Aim	Study design	Participants characteristics: nurse managers				Main relevant findings
					Size, position	Sex (female %)	Age	Educational level	
1	Bai et al. (2017) [15]	China	To explore the challenges and potential solutions to develop financial competencies	Descriptive exploratory qualitative design	18 managers who are head nurses, nurse coordinators, nurse executives, and vice presidents	100%	45.5 (range: 36–56)	NR	• Participants confronted four challenges: (1) insufficiencies in intrinsic motivation, (2) training and education, (3) cooperation and communication, and (4) insufficient reference managerial tool

2	Blaney and Hobson (1988) [16]	United States	To develop, present, and evaluate a continuing education program for nurse managers	One‐group pretest–post‐test design	36 managers who are head nurses and nursing administrators	100%	38.4	NR	• Following all‐day education program, there was a statistically significant difference between the pretest and post‐test scores (*p* < 0.01).

3	Naranjee et al. (1996) [17]	United States	To explore the economic awareness of staff nurses in which the nurse manager does and does not have budgetary responsibility	Mixed method study	41 staff nurses who had previous management experience	94%	30.6 (range: 21–54)	BSN or below (*n* = 63), MSN or above (*n* = 3), and missing (*n* = 2)	• Participants holding certifications (e.g., in clinical practice or nurse administration) scored significantly higher than those without certifications (*p* < 0.05). • Participants confronted three challenges: (1) lacked the necessary financial information and tool, (2) inability to monitor line use, and (3) absence of incentives for cost saving

4	Carruth and Carruth (2001) [18]	United States	To analyze whether nurse managers perceive performance evaluation as inclusive of financial management expectations.	Analytical cross‐sectional study	120 managers who are clinical nurse managers, supervisors, and nurse executives	89.1%	NR	NR	• Clinical supervisors participated less than other types of managers in budget preparation, planning and control, and manipulation of accounts (*p* < 0.05). • Clinical nurse managers showed greater participation in budget preparation and manipulation of accounts compared to other types of managers (*p* < 0.05). • Nurse executives participated more than other types of managers in planning and control, investigating budget variance, and manipulation of accounts (*p* < 0.05).

5	Carruth et al. (2000) [19]	United States	To determine their budgetary involvement	Descriptive cross‐sectional study	109 nurse mangers	89.8%	43.5 (range: 27–68)	BSN or below (*n* = 96), MSN or above (*n* = 13)	• Fewer than 50% of supervisors and assistant DONs helped to prepare budgets. • 65.8% of departmental directors used budgets for a mechanism for planning and control, and each unit’s needs and contribution. • Fewer than 18% of unit‐based coordinators, supervisors, and assistant DONs.

6	Consolvo and Peters (1991) [20]	United States	To provide financial management education	Descriptive cross‐sectional study	18 nurse unit managers	NR	NR	NR	• Following 3 days course, participants showed positive responses regarding leadership styles, motivation, adaptability to change, and understanding of concrete financial information.

7	Johnson (1987) [21]	United States	To determine the fiscal preparation essential for nurse administrators	Descriptive cross‐sectional study	226 nursing executives	NR	NR	BSN or below (*n* = 86), MSN or above (*n* = 137)	• The majority of participants reported receiving fiscal management education primarily through on‐the‐job training, with additional learning through self‐study and continuing education. • Participants ranked the forms of financial management education most important to nurse executives in the following order, from most to least: college coursework, on‐the‐job training, continuing education, and self‐study.

8	McFarlan (2020) [22]	United States	To examine the outcomes of an educational intervention to increase financial competency	One‐group pretest–post‐test design	23 nurse managers	100%	NR	BSN or below (*n* = 19), MSN or above (*n* = 4)	• Following the 2‐month education program, scores improved significantly both immediately postintervention (*p* < 0.05) and at 6 months postintervention (*p* < 0.05).

9	Naranjee et al. (2019) [23]	South Africa	To explore the financial management roles of nurse managers	Descriptive exploratory qualitative design	6 nurse managers	NR	NR	NR	• Financial management roles and activities of nurse managers: Nurse managers are not directly involved in the financial management of the hospitals, yet they play a contributing role in financial functioning by engaging in strategic planning, procurement, staffing, and expenditure control within their departments. • Current financial management competencies of nurse managers: (1) mostly learn through on‐the‐job experience; lack formal training, (2) limited involvement in financial tasks; nursing education focuses on clinical care • Financial management development needs: budgeting, financial reports, and legislation

10	Naranjee et al. (2019) [17]	South Africa	To explore the financial management roles of nurse managers and to develop a financial management framework	Descriptive exploratory qualitative design	6 nurse managers	NR	NR	NR	• Educational preparation for financial management in nursing programs: (1) formal educational preparation for financial management in nursing programs, (2) inadequate financial management training in basic and postbasic nursing programs • Guidance and training for financial management role: (1) informal financial management training for the current role, (2) poor support from the department of health for the current financial management role, and (3) peer support and mentoring for the financial management role

11	Ntlabezo et al. (2004) [24]	South Africa	To identify perceptions of nurse mangers regarding cost‐containment issues	Descriptive cross‐sectional study	300 of nurse unit managers, managers who engaged in supervisory activities, and chief professional managers	NR	NR	NR	• Few respondents felt adequately prepared for key financial management tasks: 6.6% for hospital budget preparation, 6.2% for manpower budget, and 10.7% for cost‐containment principle. This suggests that inadequate preparation may lead to insufficient awareness and consequently hinder effective cost management. • 69.9% of respondents indicated that nurse managers should be involved in the planning of the hospital budget. Additionally, 94.7% agreed that information regarding increases in consumable costs across units and departments should be provided to top management. • 41.3% and 18.9% felt adequately prepared for ordering and purchasing supplies and new equipment

12	Paarima et al. (2021) [25]	Ghana	To examine the financial management skills of nurse managers at the unit level	Analytical cross‐sectional study	121 nurse unit managers	73.5%	38.8 (range: 20–39)	BSN or below (*n* = 112), MSN or above (*n* = 9)	• The perceived importance of financial management competency averaged 3.19 and 2.64 on a four‐point scale. • Among the subdomains, competencies related to financial resource procurement (2.99/2.88) and financial monitoring (2.98/2.89) were perceived as the least important. • Training in management significantly contributed to the financial skills of nurse managers (*p* < 0.05).

13	Poteet et al. (1991) [26]	United States	To describe the financial responsibility of chief nurse executive	Descriptive cross‐sectional study	359 nurse managers	NR	NR	NR	• Participants indicated on‐the‐job experience (93.9%) and self‐study (59.9%) as key contributors to financial management competency. • 52% of respondents reported that no financial management education programs were available. 55% of the institutions in the study had head nurses eligible to attend financial management courses, while 81% provided financial support for some form of financial management education.

14	Welch (2022) [27]	United States	To assess the influence on unit‐based nurse managers’ self‐perception of business and financial competence in patient care	Analytical cross‐sectional study	135 RNs with formal nurse manager roles	81.4%	NR	BSN or below (*n* = 95), MSN or above (*n* = 35)	• Graduate programs provided more in‐depth information related to accounting principles (*p* < 0.1) and healthcare policy concerning quality, access, and costs (*p* < 0.05). • Managers with an MSN degree felt more confident in managing the business aspects of patient care compared to those with a BSN or ADN degree (*p* < 0.05).

*Note:* BSN: Bachelor of Science in Nursing. MSN: Master of Science in Nursing.

Abbreviations: DON, directors of nursing; NR, not reported; RN, registered nurse.

### 2.5. Collating, Summarizing, and Reporting the Results

The data underwent a two‐stage thematic analysis, in which the first stage involved inductively coding and categorizing extracted data to identify recurring patterns related to barriers, and the second stage involved synthesizing these patterns into actionable, evidence‐based strategies that can be implemented in nursing administration practice.

## 3. Results

The 14 studies that met the inclusion criteria were conducted in the following countries: China (*n* = 1), Ghana (*n* = 1), South Africa (*n* = 3), and the USA (*n* = 9). The study designs were analytical cross‐sectional (*n* = 8), qualitative (*n* = 3), experimental (*n* = 2), and mixed methods (*n* = 1). The total number of participants across all studies was 1518 people. Of these, 176 were registered nurses (RNs) who had previous management experience. The remaining participants were currently working as nurse managers. Regarding managerial classifications, eight studies specified the participants’ positions, seven did not specify positions, and one provided classifications without indicating the number of participants per role. The positions identified were 731 nurse managers without specific titles, 262 nurse unit managers (or clinical nurse managers), 251 nurse executives, 176 RNs, 39 department/division directors, 31 supervisors, 15 DONs, 8 assistant DONs, 3 nurse coordinators, and 2 vice presidents.

Gender data were reported in eight studies, with 545 female nurse managers and 76 male nurse managers identified. The mean participant age was 39.3 years (range: 21–68 years). Educational background was available in seven studies, with 571 participants holding a BSN or less and 217 holding an MSN or more. The 14 included studies spanned multiple decades from the 1980s to the 2020s.

We identified a total of six subthemes regarding financial management among nurse managers: three factors hindering financial management and three coping strategies to improve financial management (Table 2). The three factors hindering financial management among nurse managers were defined as (1) insufficient awareness [19, 24, 25], (2) lack of authority [15, 18, 24, 28], and (3) inadequate resources and systems [17, 19, 24]. The three key coping strategies that emerged from the literature for addressing the challenges of financial management were (1) practical experience [21, 23, 26, 28], (2) professional credentials [17, 21, 27], and (3) targeted training and educational programs [16, 19, 20, 22, 25, 26, 28].

**TABLE 2:** Themes identified within the included articles.

Main themes	Subthemes	Articles
Factors hindering financial management	Insufficient awareness	[19, 24, 25]
	Lack of authority	[15, 18, 24, 28]
	Inadequate resources and systems	[17, 19, 24]

Strategies for improving financial management	Practical experience	[21, 23, 26, 28]
	Professional credentials	[17, 21, 27]
	Targeted training and educational programs	[16, 19, 20, 22, 25, 26, 28]

### 3.1. Factors Hindering Financial Management

#### 3.1.1. Insufficient Awareness

Research has shown that nurse managers have low levels of readiness in core financial tasks, with only a small percentage feeling prepared for hospital budgeting (6.6%), workforce budgeting (6.2%), or understanding cost‐containment principles (10.7%), all of which suggests low awareness of financial responsibilities [24]. In a study employing the instrument developed by Chase [29], their perceived importance of knowledge and ability related to financial management yielded mean scores of 3.19 and 2.64 out of 4, respectively [25]. The study indicated that these moderate scores reflect a lack of sufficient financial management skills among them [25].

Similarly, nurse unit managers—a subset of nurse managers responsible for ward‐level operations—have demonstrated relatively low awareness of the importance of financial management, often perceiving themselves more as operational implementers than as individuals directly accountable for financial outcomes [19]. This perception stands in contrast to the expectations of higher‐level nurse leaders, who regard financial management as a pivotal responsibility for nurse unit managers that is essential for achieving both economic and organizational benefits [15].

#### 3.1.2. Lack of Authority

Although 69.9% of nurse managers support their involvement in budget planning and 94.7% believe that top management should be informed of cost increases [24], their actual authority over financial matters remains both limited and inconsistent across roles and institutions. The level of financial authority varies by position. Those without direct financial authority often participate in departmental budget meetings without contributing to actual decision‐making processes, which in turn limits their opportunities to develop financial competency [28]. In one study [15], only 18% of nurse unit managers and other midlevel managers (e.g., supervisors and assistant DONs) were involved in correcting budget variances and fewer than 50% participated in budget preparation. In contrast, more than half of departmental directors and DONs were engaged in correcting budget variances, and 65.8% of departmental directors used budgets as a tool for planning and control that reflects the specific needs and contributions of each unit [15]. Similar results were found in another study, where clinical nurse managers were most frequently engaged in budget preparation and account management (*p* = 0.016), while nurse executives were more active in financial planning, control, and variance investigation (*p* < 0.05) [18]. Across studies, financial authority and involvement in budget‐related activities varied by hierarchical position, with higher engagement observed among departmental directors and DONs compared to midlevel and lower‐level managers.

#### 3.1.3. Inadequate Resources and Systems

Nurse managers lack sufficient operational tools such as finance‐related applications and up‐to‐date reference materials, which are necessary for effective financial management [15]. Their perceived readiness for managing the financial aspects of supplies and equipment is low, with 41.3% feeling prepared for supply management and 18.9% feeling prepared for equipment management [24]. In addition, access to financial and budget data varies significantly between institutions [17]. While some hospitals provide regular, detailed, and analytic reports covering various expenditure categories, others offer only minimal or on‐demand data [28]. In some cases, no financial information is shared at all, making it difficult for them to monitor and assess their unit’s budgetary performance [17].

The lack of a structured financial assessment system in nursing further complicates this issue. Limited—and mostly punitive—indicators are used, and there are no established mechanisms to track key operational factors such as linen usage, all of which reflects a broader absence of supportive management systems [17, 19].

### 3.2. Strategies for Improving Financial Management

#### 3.2.1. Practical Experience

Practical experience is consistently identified as a critical pathway through which nurse managers develop financial skills. In a study of 359 nurse managers, 94% indicated that hands‐on experience contributed to their ability to manage finances, particularly in areas such as accounting, computing, and staffing/scheduling [26]. Similarly, a study of 226 nursing executives reported that most had gained their financial competency primarily through on‐the‐job experience rather than formal education [21]. These executives noted that they had to acquire essential knowledge in calculating full‐time equivalents, costing services, analyzing financial reports, and understanding reimbursement systems while actively engaged in their roles. Further supporting this view, interviews with six nurse managers revealed that most of their financial competencies were developed during their day‐to‐day responsibilities rather than through prior training [23]. They developed financial planning skills on the job by participating in strategic management workshops, linking operational plans to budgets, creating procurement plans, managing nursing human resources, contributing to budget development, and monitoring financial performance through report reviews [28]. Across studies, on‐the‐job experience emerged as the predominant mode of financial competency development, often in the absence of formal training.

#### 3.2.2. Professional Credentials

Professional credentials—including academic degrees and clinical or administrative certifications—have been associated with nurse managers’ financial management competencies. According to Caroselli [17], nurses with professional certifications in areas such as clinical practice or nurse administration scored significantly higher in financial competency assessments compared to those without such credentials.

Educational background was also associated with financial competency. Nurses holding a BSN or an MSN reported greater exposure to healthcare accounting, finance and reimbursement, budgeting, and quality‐related policy during their undergraduate studies compared to those with an ADN, and graduate programs provided a more in‐depth coverage of accounting principles and healthcare policy [27]. Additionally, those with an MSN degree reported significantly greater confidence in managing the business aspects of patient care compared to those with a BSN or ADN (*p* < 0.05) [27]. In contrast, Johnson [21] found that nursing executives had received financial management training through various means, more commonly through self‐study, continuing education, and on‐the‐job training than through formal college coursework, suggesting that formal academic credentials alone may not fully account for the development of financial competency in practice.

#### 3.2.3. Targeted Training and Educational Programs

The need for formal financial training and education for nurse managers has been recognized [19, 26, 28]. Essential topics include budgeting, financial report interpretation, relevant legislation, cost center management, and structured training program development [18]. Studies have shown that financial management training improves nurse managers’ knowledge significantly [16, 20, 22, 25]. Core training topics often cover basic financial terminology, budgeting and variance analysis, staffing and resource planning, linking clinical practice to financial outcomes, healthcare revenue structures, and organizational management strategies such as change management and turnover reduction [16, 20, 22].

## 4. Discussion

This study aimed to explore the challenges and potential strategies related to improving nurse managers’ financial management competencies. To this end, we conducted a scoping review that synthesized findings from multiple contexts, study designs, and participant groups. From the literature, we identified three key challenges: insufficient awareness, lack of authority, and inadequate resources and systems. To address these, three strategic approaches were identified: acquiring practical experience, obtaining professional credentials, and developing and attending targeted training and education programs.

Insufficient awareness refers to the lack of perceived importance that nurse managers place on financial management, which was cited as a significant barrier to their effectiveness in this area. According to prior studies, nurse managers agree that understanding financial management helps improve resource allocation in the public health sector [30]. However, they report a lack of confidence in areas such as monitoring unit budgets, understanding healthcare finance policies, interpreting financial reports, and implementing cost reduction strategies [30]. These difficulties are further exacerbated by systemic issues such as insufficient education, a lack of intrinsic motivation, and increasing demands on resources [3, 15]. Unlike earlier studies that framed financial awareness primarily as an individual deficit [21, 28], more recent evidence suggests that insufficient awareness is also shaped by organizational and structural factors, implying that institutional‐level interventions are equally necessary [3, 4]. Given that structured exposure, experiential learning, and contextual discussions provided through workshops have been shown to improve financial competency [30], it is crucial for healthcare organizations to focus on raising awareness and providing systematic support to address these barriers.

Institutional structures including the delegation of financial authority to nurse managers across broader positions are fundamental to strengthening nurse managers’ financial roles. Variability in managerial titles—such as nurse unit manager versus nurse executive—often reflects differences in delegated authority [17–19], which directly influences nurse managers’ opportunities to develop financial management competencies. The extent of the financial management authority exercised by nurse managers has been found to vary according to the institutional context and managerial position. For instance, nurse managers in nursing homes and community health centers have greater financial autonomy and decision‐making authority and thus require greater skills in financial negotiation, compared to nurse managers in hospitals [10, 31]. This variability in delegated authority across settings is further supported by Ismail et al. [4], who found that nurse leaders in hospital settings frequently reported financial decisions being controlled exclusively by hospital administrators, which restricted their ability to distribute resources according to patient needs. This finding is consistent with Macinati and Rizzo [31], who demonstrated that clinical managers with greater budgetary involvement reported higher performance outcomes, suggesting that delegated authority is not merely a structural feature but a driver of competency development. Limited decision‐making authority and financial constraints can lead to inefficient resource allocation and frustration among nurse managers [4]. These frustrations have also been linked to increased turnover intention [10], highlighting the broader organizational cost of failing to delegate financial authority appropriately, both of which have been identified as key factors contributing to their resignation [10]. Based on the findings of this study, Healthcare organizations should instead work to standardize the delegation of financial authority and ensure access to appropriate financial tools and systemic support tailored to their institutional and managerial contexts. As an example, a stepwise delegation model that gradually involves nurse managers in budget planning to improve their preparedness for equipment management could help them develop financial management competencies in a practical and meaningful way. Such organizational and policy‐level initiatives are essential to empower nurse managers to fulfill their financial and managerial responsibilities effectively.

In addition to delegating financial authority, it is essential to ensure that nurse managers have access to appropriate financial management resources and systemic support for effective performance in their roles. A resource‐rich environment enables nurse managers to effectively demonstrate their managerial competencies [32]. On the other hand, hospital regulations that are implemented without considering clinical realities can increase nurse managers’ workload due to systemic deficiencies [33]. Integrating adequate material resources and effective systemic support is essential for nurse managers to overcome financial constraints and enhance their managerial competencies [4, 15]. This challenge is not limited to financial resources alone. Evidence from Finland indicates that current information systems do not effectively support nurse managers’ duties in reporting, resource management, and performance assessment and that managers are frequently required to collect data from multiple fragmented systems [44]. Similarly, Chinese head nurses have identified the lack of adequate managerial tools and information support as a significant barrier to effective financial management practice [15]. Taken together, these findings suggest that the challenge of inadequate resources and systems is not simply a matter of budget allocation, but reflects a broader gap in organizational infrastructure that must be addressed at the policy level. There is a critical need for organizational and policy‐level efforts to establish clear delegation of financial authority and to provide nurse managers with the resources and systemic support that will enable them to perform their financial management roles effectively.

Practical experience was recognized as the primary strategy through which nurse managers acquire financial management competencies. Notably, those with less than 3 years of managerial experience demonstrate lower financial knowledge compared to those with over 4 years of experience [34]. Despite their practical experience, however, many nurse managers report feeling underprepared for their financial responsibilities [30]. This sense of unpreparedness is not unique to a specific context; similar findings have been reported across diverse healthcare settings, including Ghana [25], South Africa [27], and the USA [27], suggesting that the gap between practical experience and financial competency is a widespread, systemic issue rather than an individual one. A significant number had not completed any formal finance‐related courses prior to transitioning into leadership roles [15]. This suggests that relying solely on nurse managers’ practical experience to develop essential financial management skills has obvious limitations. Additionally, this scoping review found variability in financial competency levels among individual nurse managers, with those holding a master’s degree tending to exhibit greater financial literacy [4, 30]. Even within the same role, however, individual differences in financial management abilities have been observed [3], indicating that educational approaches should be tailored to competency levels (e.g., novice, competent practitioner, and expert). Recognizing this variability underscores the need for both institutional support and targeted educational interventions.

Key financial management competencies necessary for nursing practice include financial analysis, balance sheets, income statements, concepts and analysis of financial ratios, and legislation [18, 35]. These components should be incorporated into educational content. For ongoing professional development, formal training should be provided through systematic programs alongside web‐based platforms; it should include instruction on the use of financial support tools [36]. Incorporating practical approaches such as on‐site workshops with simulations, case‐based learning, and coaching programs in collaboration with hospital finance teams can enhance educational effectiveness [37–39]. To further enhance effectiveness, using performance indicators to evaluate training outcomes can strengthen the applicability of financial management skills to clinical practice [40]. These approaches align with evidence from broader healthcare management education, which indicates that multimodal training combining didactic and experiential methods yields stronger and more sustained competency gains than single‐format programs [38].

Establishing an educational framework specifically to enhance financial management competencies would address the current gaps in formal training and skill development [3]. The development of structured, competency‐based financial education programs is therefore recommended, as it would not only fill existing competency gaps but also strengthen nurse managers’ capacity to contribute to effective and accountable healthcare delivery. It has been noted that nurse managers often fail to recognize the importance of financial management, while assessing their own financial management competencies as low [41, 42]. Targeted educational programs should thus focus on raising awareness alongside skill development.

Educational interventions alone, however, may not be sufficient. Without supportive infrastructures and clear connections to patient care, even well‐designed educational programs may fail to produce meaningful practice improvements. Thus, our findings also highlight the need for broader organizational changes to support nursing management practice. Organizations must reconsider how financial authority is distributed and integrate user‐friendly financial systems with structured partnerships between nursing and finance departments. Most importantly, linking financial management directly to patient outcomes could help bridge the gap between administrative responsibilities and clinical priorities, as nurse managers’ engagement in administrative roles has been associated with higher patient safety culture awareness [43]. In fact, combining targeted education with organizational reform will be essential for improving both cost management and patient safety.

Taken together, these findings have important implications for both practice and policy, suggesting that strengthening financial competency in nurse managers can directly enhance organizational accountability and ultimately contribute to safer, more sustainable patient care.

### 4.1. Limitations and Strengths

While we performed a comprehensive review of literature from the 1980s through the 2020s to explore the financial management of nurse managers, only 14 studies were ultimately included in the review. This limited our ability to conduct comparative analyses across different time periods or policy changes. The heterogeneity in research designs and focal topics among these studies also necessitates caution in interpreting the findings. The overall scarcity of research on this topic highlights the need for more robust theoretical frameworks and empirical investigations in this area.

Despite the limitations, the present study sheds light on the challenges nurse managers face in financial management and identifies strategic approaches to address them. By highlighting the need for both structural reform—such as authority delegation and provision of resources—and individualized educational support, this study offers meaningful practical implications. The findings can inform the development of institutional policies and training programs that are responsive to varying managerial roles and competencies. Future research should explore the long‐term effects of tailored education and structural reforms on nurse managers’ financial management competencies and job performance through longitudinal studies. In addition, studies examining how organizational culture and leadership styles influence the delegation of financial authority and resource access could offer valuable insights. Qualitative investigations into nurse managers’ lived experiences would help to identify context‐specific barriers and inform more targeted policy and practice strategies.

## 5. Conclusion

This scoping review examined the challenges nurse managers face and the strategies they employ to enhance their financial management competencies. A total of six subthemes were identified—three factors hindering financial management among nurse managers and three coping strategies for improving their financial management competencies. The key challenges were insufficient individual awareness, lack of authority, and inadequate resources and systems. In response to these challenges, three strategic approaches emerged: gaining practical experience, obtaining professional credentials, and participating in targeted education and training programs. The findings highlight that both institutional structures and educational efforts are necessary to support nurse managers in fulfilling their financial responsibilities. These insights provide practical and policy‐level implications for enhancing financial management competencies in nursing leadership. As the evidence base in this area continues to grow, integrating financial management competency development into nursing leadership policy and curricula will be essential for building a financially prepared nursing workforce.

## Author Contributions

Jiae Lee: conceptualization, methodology, investigation, data curation, formal analysis, writing–original draft, and writing–review and editing. Yoonjung Kim: methodology, data curation, formal analysis, and writing–review and editing. Eunjin Kim: methodology, investigation, data curation, formal analysis, and writing–review and editing.

## Funding

The authors declare that they did not receive any financial support for conducting this study.

## Conflicts of Interest

The authors declare no conflicts of interest.

## Data Availability

Data sharing is not applicable to this article as no new data were created or analyzed in this study. This scoping review synthesizes findings from previously published research, all of which are cited and publicly accessible.

## References

[1] Glied S. and Chandra A. , It is Time to Consider More Price Regulation in Health Care, JAMA Health Forum. (2024) 5, no. 6, 10.1001/jamahealthforum.2024.2342. 38869886

[2] Meyers R. C. and Kottapalli P. , Strategic Planning and Aggressiveness in Healthcare: Navigating Uncertainty for Organizational Success, Healthc Strategy Rev. (2025) 1, no. 3, 42–54, 10.61449/ajhcs.2025.9.

[3] Bayram A. , Pokorná A. , Ličen S. et al., Financial Competencies as Investigated in the Nursing Field: Findings of a Scoping Review, Journal of Nursing Management. (2022) 30, no. 12, 2801–2810, 10.1111/jonm.13671. 35538845 PMC10084091

[4] Ismail H. A. , Kotp M. H. , Basyouny H. A. A. et al., Empowering Nurse Leaders: Leveraging Financial Management Practices to Foster Sustainable Healthcare—a Mixed-Methods Study, BMC Nursing. (2025) 24, no. 1, 10.1186/s12912-025-02981-6. PMC11951552 40156004

[5] Akinleye D. D. , McNutt L. A. , Lazariu V. , and McLaughlin C. C. , Correlation Between Hospital Finances and Quality and Safety of Patient Care, PLoS One. (2019) 14, no. 8, 10.1371/journal.pone.0219124, 2-s2.0-85070756377. PMC6697357 31419227

[6] American Nurses Association , Financial Management Skills for Nurse Managers, 2023, https://www.nursingworld.org/content-hub/resources/nursing-leadership/nursing-financial-management/.

[7] Abu Arra A. Y. , Ayed A. , Toqan D. et al., The Factors Influencing Nurses’ Clinical Decision-Making in Emergency Department, Inquiry: The Journal of Health Care Organization, Provision, and Financing. (2023) 60, 10.1177/00469580231152080. PMC9893345 36705018

[8] Batran A. , Al-Humran S. M. , Malak M. Z. , and Ayed A. , The Relationship Between Nursing Informatics Competency and Clinical Decision-Making Among Nurses in West Bank, Palestine, CIN: Computers, Informatics, Nursing. (2022) 40, no. 8, 547–553, 10.1097/CIN.0000000000000890. 35234705

[9] Daly J. , Jackson D. , Mannix J. , Davidson P. M. , and Hutchinson M. , The Importance of Clinical Leadership in the Hospital Setting, Journal of Healthcare Leadership. (2014) 6, 75–83, 10.2147/JHL.S46161, 2-s2.0-84943266893.

[10] Erjavec K. and Starc J. , Competencies of Nurse Managers in Slovenia: A Qualitative and Quantitative Study, Central European Journal of Nursing and Midwifery. (2017) 8, no. 2, 632–640, 10.15452/CEJNM.2017.08.0012, 2-s2.0-85020281370.

[11] Pollock D. , Peters M. D. J. , Khalil H. et al., Recommendations for the Extraction, Analysis, and Presentation of Results in Scoping Reviews, JBI Evidence Synthesis. (2023) 21, no. 3, 520–532, 10.11124/JBIES-22-00123. 36081365

[12] Arksey H. and O’Malley L. , Scoping Studies: Towards a Methodological Framework, International Journal of Social Research Methodology. (2005) 8, no. 1, 19–32, 10.1080/1364557032000119616, 2-s2.0-14644388070.

[13] Tricco A. C. , Lillie E. , Zarin W. et al., PRISMA Extension for Scoping Reviews (PRISMA-ScR): Checklist and Explanation, Annals of Internal Medicine. (2018) 169, no. 7, 467–473, 10.7326/M18-0850, 2-s2.0-85054287365. 30178033

[14] Page M. J. , McKenzie J. E. , Bossuyt P. M. et al., The PRISMA 2020 Statement: An Updated Guideline for Reporting Systematic Reviews, British Medical Journal. (2021) 372, 10.1136/bmj.n71. PMC8005924 33782057

[15] Bai Y. , Gu C. , Chen Q. , Xiao J. , Liu D. , and Tang S. , The Challenges that Head Nurses Confront on Financial Management Today: A Qualitative Study, International Journal of Nursing Science. (2017) 4, no. 2, 151–157, 10.1016/j.ijnss.2017.03.007, 2-s2.0-85017149469. PMC6626139 31406731

[16] Blaney D. R. and Hobson C. J. , Developing Financial Management Skills: An Educational Approach, Journal of Nursing Administration. (June 1988) 18, no. 6, 13–16, 10.1097/00005110-198806010-00005. 3131502

[17] Naranjee N. , Sibiya M. N. , and Ngxongo T. S. P. , Development of a Financial Management Competency Framework for Nurse Managers in Public Health Care Organisations in the Province of KwaZulu-Natal, South Africa, International Journal of Africa Nursing Sciences. (2019) 14, 10.1016/j.ijans.2019.100154, 2-s2.0-85069920440.

[18] Carruth P. J. and Carruth A. K. , A Comparative Analysis of the Budget-Related Activities Used in the Evaluation of Clinical Nurse Managers, Clinical Supervisors and Nurse Executives, Research in Healthcare Financial Management. (2001) 6, no. 1, 91–103.

[19] Carruth A. K. , Carruth P. J. , and Noto E. C. , Nurse Managers Flex Their Budgetary Might, Nursing Management. (2000) 31, no. 2, 16–17, 10.1097/00006247-200002000-00016, 2-s2.0-0034137927. 10827687

[20] Consolvo C. A. and Peters M. , Financial Management for Nurse Managers—The Bottom Line for Renewal, The Journal of Continuing Education in Nursing. (1991) 22, no. 6, 245–247, 10.3928/0022-0124-19911101-06. 1955591

[21] Johnson M. S. , Preparing Nurse Executives for Financial Management, Nursing Administration Quarterly. (1987) 12, no. 1, 67–73, 10.1097/00006216-198701210-00013, 2-s2.0-0023409224. 3684003

[22] McFarlan S. , An Experiential Educational Intervention to Improve Nurse Managers’ Knowledge and Self-Assessed Competence With Health Care Financial Management, The Journal of Continuing Education in Nursing. (2020) 51, no. 4, 181–188, 10.3928/00220124-20200317-08. 32232494

[23] Naranjee N. , Ngxongo T. S. P. , and Sibiya M. N. , Financial Management Roles of Nurse Managers in Selected Public Hospitals in KwaZulu-Natal Province, South Africa, African Journal of Primary Health Care and Family Medicine. (2019) 11, no. 1, 10.4102/phcfm.v11i1.1981, 2-s2.0-85072757262. PMC6779971 31588771

[24] Ntlabezo E. T. , Ehlers V. J. , and Booyens S. W. , South African Nurse Managers’ Perceptions Regarding Cost Containment in Public Hospitals, Curationis. (August 2004) 27, no. 3, 34–42, 10.4102/curationis.v27i3.996. 15777028

[25] Paarima Y. , Kwashie A. A. , and Ofei A. M. A. , Financial Management Skills of Nurse Managers in the Eastern Region of Ghana, International Journal of Africa Nursing Sciences. (2021) 14, 10.1016/j.ijans.2020.100269.

[26] Poteet G. W. , Hodges L. C. , and Goddard N. , Financial Responsibilities and Preparation of Chief Nurse Executives, Nursing Economics. (September-October 1991) 9, no. 5, 336–341. 1922437

[27] Welch T. D. , Do Nurse Managers Feel Competent in the Financial and Business Aspects of Their Roles? Exploring Self-Perceptions, Journal of Nursing Administration. (May 2022) 52, no. 5, 286–292, 10.1097/NNA.0000000000001149. 35467594

[28] Caroselli C. , Economic Awareness of Nurses: Relationship to Budgetary Control, Nursing Economics. (1996) 14, no. 5, 292–296. 8998024

[29] Chase L. K. , Nurse Manager Competencies, 2010, University of Iowa, doctoral dissertation.

[30] Zuma K. and Mahomed O. , Financial Management Literacy Among Nurse Managers in Two Districts of KwaZulu-Natal, South Africa: A Cross-Sectional Study, International Journal of Africa Nursing Sciences. (2025) 23, 10.1016/j.ijans.2025.100862.

[31] Macinati M. S. and Rizzo M. G. , Exploring the Link Between Clinical Managers’ Involvement in Budgeting and Performance: Insights From the Italian Public Health Care Sector, Health Care Management Review. (2016) 41, no. 3, 213–223, 10.1097/HMR.0000000000000071, 2-s2.0-84977536143. 26052784

[32] Lee R. , Kim M. , Choi S. , and Shin H. Y. , Factors Influencing Managerial Competence of Frontline Nurse Managers, Journal of Korean Academy of Nursing Administration. (2018) 24, no. 5, 435–444, 10.11111/jkana.2018.24.5.435, 2-s2.0-85063615660.

[33] Moon J. H. , Joo G. E. , and Lee J. , Grounded Theoretical Analysis on the Hospital Accreditation Experience of Head Nurses in General Hospitals, Journal of Korean Academy of Nursing Administration. (2016) 22, no. 5, 437–447, 10.11111/jkana.2016.22.5.437.

[34] Wong Q. , An Educational Program to Elevate the Financial Acumen of Nurse Leaders, Nursing Management. (2024) 55, no. 4, 12–20, 10.1097/nmg.0000000000000111. 38557747

[35] Lim J. Y. , Noh W. , Oh S. E. , and Kim O. G. , Financial Ratio Analysis for Developing Nursing Management Strategies in University Hospitals, Journal of Korean Academy of Nursing Administration. (2013) 19, no. 1, 7–16, 10.11111/jkana.2013.19.1.7.

[36] Chetty D. R. , Du Plessis A. H. , Ham-Baloyi W. , Naidoo J. , and van Rooyen D. R. M. , Facilitating Utilization of Evidence-Informed Management by Nurse Managers in Healthcare Facilities: An Integrative Literature Review, Journal of Nursing Management. (2024) 2024, 10.1155/2024/6649401. PMC11919202 40224871

[37] Thistlethwaite J. E. , Davies D. , Ekeocha S. et al., The Effectiveness of Case-Based Learning in Health Professional Education. A BEME Systematic Review: BEME Guide No. 23, Teaching and Learning in Medicine. (2012) 24, no. 1, e421–e444, 10.3109/0142159X.2012.680939, 2-s2.0-84861038728. 22578051

[38] Ravaghi H. , Beyranvand T. , Mannion R. , Alijanzadeh M. , Aryankhesal A. , and Belorgeot V. D. , Effectiveness of Training and Educational Programs for Hospital Managers: A Systematic Review, Health Services Management Research. (2021) 34, no. 2, 81–91, 10.1177/0951484820971460. 33143488

[39] Richardson C. , Wicking K. , Biedermann N. , and Langtree T. , Coaching in Nursing: An Integrative Literature Review, Nursing Open. (2023) 10, no. 4, 1925–6649, 10.1002/nop2.1925. PMC10495732 37365717

[40] Ghofrani M. , Valizadeh L. , Zamanzadeh V. , Ghahramanian A. , Janati A. , and Taleghani F. , Baccalaureate Nursing Education Institutions’ Key Performance Indicators: A Review of the Existing Indicators and Qualitative Analysis of Expert Interviews, BMC Nursing. (2023) 22, no. 1, 10.1186/s12912-023-01484-6. PMC10552274 37798710

[41] Lee J. and Kim M. , Factors Impacting on Nurse Unit Managers’ Knowledge and Ability Importance of Managerial Competencies, Journal of Korean Academy of Nursing Administration. (2024) 30, no. 4, 428–438, 10.11111/jkana.2024.30.4.428.

[42] Ben Natan M. and Har Noy R. , Required Competencies for Nurse Managers in Geriatric Care: The Viewpoint of Staff Nurses, International Journal of Caring Sciences. (September-December 2016) 9, no. 3.

[43] Malak M. Z. , Salouk J. , Al-Shawawreh R. , Al-Kamiseh H. , and Ayed A. , Perceptions of Patient Safety Culture Among Emergency Room Nurses in Jordanian Accredited Hospitals, Journal of Nursing Management. (2022) 30, no. 7, 3131–3138, 10.1111/jonm.13729. 35765702

